# Epidemiology and mortality predictors for severe childhood community-acquired pneumonia in ICUs: A retrospective observational study

**DOI:** 10.3389/fped.2023.1031423

**Published:** 2023-03-23

**Authors:** Lu Cao, Zhaohua Ji, Peng Zhang, Jingwen Wang

**Affiliations:** ^1^ Department of Pharmacy, Shaanxi Provincial People's Hospital, Xi'an, China; ^2^ Department of Epidemiology and the Ministry of Education Key Lab of Hazard Assessment and Control in Special Operational Environment, School of Public Health, Fourth Military Medical University, Xi'an, China; ^3^ Department of Pharmacy, Xijing Hospital, Fourth Military Medical University, Xi'an, China

**Keywords:** pneumonia, children, pediatric intensive care unit, mortality, risk factors

## Abstract

**Background:**

To identify the epidemiology and mortality predictors of severe childhood community-acquired pneumonia (CAP) and evaluate the influence of medications on clinical outcomes in the real world.

**Methods:**

We conducted a multicenter retrospective observational study among children aged ≤5 years with severe CAP, separately comparing the detailed information between those who experienced in-hospital death and those who survived in three different age groups. A multivariate logistic regression model was used to determine mortality predictors.

**Results:**

A total of 945 children were recruited: 341 young children aged 2–59 months, 47 infants aged 29 days to 2 months, and 557 neonates aged less than 28 days. A total of 88 deaths occurred (9.3%). There was low adherence to antimicrobial guidelines in the group aged 2–59 months, and carbapenems widely served as initial empirical regimens. However, analysis of all three age groups showed that the efficacy of antibacterial drugs with initial empirical selection grades higher than those recommended by the guidelines was not better than that of antibacterial drugs with grades recommended by the guidelines. In multivariate analyses, very severe pneumonia (odds ratio (OR): 3.48; 95% confidence interval (CI): 1.36–8.93), lower birth weight (OR: 4.64; 95% CI: 1.78–12.20), severe underweight (OR: 6.06; 95% CI: 2.34–15.63), mechanical ventilation (OR: 2.58; 95% CI: 1.00–6.62; OR: 15.63; 95% CI 3.25–76.92), a higher number of comorbidities (OR: 8.40; 95% CI: 1.89–37.04), comorbidities including anemia (OR: 5.24; 95% CI: 2.33–11.76) and gastrointestinal hemorrhage (OR: 3.79; 95% CI: 1.36–10.53), and the use of sedative-hypnotics (OR: 2.60; 95% CI: 1.14–5.95) were independent risk factors for death; infants treated with probiotics had a lower mortality rate (OR: 0.14; 95% CI: 0.06–0.33).

**Conclusions:**

Severe CAP remains a primary cause of death in children under 5 years of age. Clinical characteristics, comorbidities and medications are evidently associated with death. Importantly, we should pay particular attention to the identification of mortality predictors and establish prophylactic measures to reduce mortality.

## Introduction

Pneumonia is an acute infection of the lung parenchyma and/or interstitial part of the lung, causing symptoms of hypoxia and infection to different degrees, usually fever, cough, rapid breathing, moist rales, and abnormal chest x-ray changes (1). Pneumonia is the most common illness and cause of hospitalization and the leading cause of death in children under 5 years of age, especially infants (2–4), owing to immature systemic organ and immune system development, insufficient IgA secretion in their respiratory mucosa, weak cough and expectoration abilities, and poor swallowing reflexes predisposing them to reflux (5). Recent studies had shown that pneumonia caused 740,180 deaths in children younger than 5 years of age worldwide in 2019, accounting for approximately 14% of under-5 deaths (6). In addition, a systematic analysis published in 2019 demonstrated that clinical pneumonia morbidity in children younger than 5 years of age decreased from 25.9% in 2000 to 8.4% in 2015 along with the decrease in mortality from 22.6% in 1996 to 12.2% in 2015 in China; however, there were still approximately 700,000 children hospitalized with pneumonia and 22,200 children who died from pneumonia each year in China (7, 8). Therefore, it is urgent to decrease childhood pneumonia mortality, which can be achieved through early identification and the management of mortality predictors.

At present, several published studies had shown that sex, symptom duration, concurrent underweight age-related, very severe pneumonia, severe malnutrition, diarrhea, fever, organ failure index scores, congenital heart disease (CHD), and HIV infection were risk factors for severe pneumonia death in children (2, 4, 9–13). A few randomized controlled trials (RCTs) had revealed that inappropriate initial antibiotic therapy (11, 14–17) and the use of other concomitant medications, including ambroxol, probiotics, bronchodilators (18–21), and sedative-hypnotics (22) were associated with all-cause mortality in children younger than 5 years of age with CAP. Nevertheless, there is scarce information about the influence of antimicrobial practices and other concomitant medications on the clinical outcomes of severe childhood CAP in ICUs under real-world clinical conditions (23).

In this study, we collected detailed information and clinical outcome data from severe childhood CAP in PICUs at discharge, attempting to describe the etiology, epidemiological characteristics, and risk factors associated with death, especially the effects of concomitant drugs and comorbidities, to provide real-life clinical evidence for the treatment of severe childhood CAP in PICUs, thereby improving the survival rate.

## Methods

### Study design and eligibility criteria

This was a multicenter retrospective observational study of children admitted to the PICUs of two large teaching hospitals in Northwest China between January 2012 and January 2019. The inclusion criteria was children aged <5 years with severe or very severe CAP in the community, including pneumonia occurring after admission due to pathogen infection with a clear incubation period outside the hospital (1). The exclusion criteria for patients comprised (1) patients who had previously been included in other clinical trials during the same research period; (2) patients without antimicrobial treatment in the first 2 days after PICU admission (16); (3) patients with a duration of antimicrobial therapy <48 h (16); (4) patients with hospital-acquired pneumonia; (5) patients with severe congenital malformations or severe metabolic disorders; and (6) patients with imperfect data records.

Due to the classification of severe CAP, the epidemiology, diagnosis, common comorbidities, and therapeutic regimens are completely different among neonates aged less than 28 days, young infants aged 29 days to 2 months, and children aged 2–59 months; therefore, data were collected and analyzed separately for the three age groups (1, 2).

### Variables and definitions

The diagnosis of pneumonia was made by physicians according to symptoms of fever, cough, wheezing, increased respiration and moist rales, imaging features on chest x-ray or CT, and complications such as empyema, pneumothorax, acute respiratory distress syndrome (ARDS), respiratory failure, and sepsis (1, 24). Severe or very severe pneumonia was defined by the World Health Organization (WHO) classification for pneumonia severity.

We collected detailed information on the following from medical records: demographics (e.g., sex, weight-for-age category and birth weight); medical history (e.g., antimicrobial therapy use prior to admission); clinical features of pneumonia at PICU admission (fever defined as temperature >38°C (2), pneumonia severity, symptom duration ≥21 days (2), and mechanical ventilation); white blood cell (WBC) count (an abnormal WBC count defined as <5 or >15 × 10^9^/L and determined with blood samples obtained in the first 2 days of PICU hospitalization) (9, 25, 26); microbiological data (only considering the culture results from blood and cerebrospinal fluid samples); comorbidities (according to clinical diagnoses and suspicions; diseases in more than 6% of the total sample selected for analysis); initial antimicrobial therapy (defined as the antimicrobial regimens used within the first 2 days of PICU admission) (14); other concomitant medications (medications selected for use in more than 6% of the total sample, those in many reports in the literature, those with great controversies regarding their clinical use, and those concerning clinicians) used during hospitalization; and outcomes at discharge.

The WHO classification of pneumonia severity is as follows (27): (1) severe pneumonia: cough and/or difficulty breathing, with lower chest indrawing for young children aged 2–59 months and with lower chest indrawing or tachypnea for young infants; and (2) very severe pneumonia: severe pneumonia plus at least one of the following: unconsciousness, lethargy or convulsions; severe dyspnea; inability to drink or breastfeed; oxygen saturation <90% or central cyanosis; or serious complications, including heart failure, respiratory failure, shock, empyema, sepsis, and multiple organ dysfunction syndrome (MODS).

Antibacterial drugs with grades higher than those recommended by guidelines were judged according to the WHO guidelines for severe childhood pneumonia. A combination of ampicillin/penicillin and gentamicin or the use of broad-spectrum antimicrobials alone, including third-generation cephalosporins and chloramphenicol, is recommended as the first choice for young infants under 2 months of age with either severe pneumonia or very severe pneumonia and for children aged 2–59 months with very severe pneumonia (14, 16, 17). Narrow-spectrum therapies (i.e., penicillin or ampicillin) are recommended for severe pneumonia in children aged 2–59 months (14, 16, 17).

Weight-for-age categories were classified based on the 2006 WHO standards (8). Moderate underweight and undernutrition were defined as a weight-for-age ranging from −3 to −2 standard deviations (SDs) from the median, and severe undernutrition and underweight were defined as a weight-for-age <−2 SDs from the median.

### Clinical outcomes

The primary outcome evaluated in the period of our investigation was in-hospital mortality (28, 29).

### Ethics

The materials related to this research, including the protocol and “Severe Pneumonia Inpatient Recording Chart”, were submitted to and approved by the ethics committees of the research hospitals.

### Statistical analysis

The characteristics of the participants were stratified into a survivor group and a nonsurvivor group according to the outcome at discharge. All categorical variables were compared between two groups by Pearson *χ*^2^ and Fisher's exact tests. The Cochran-Armitage trend test (*Z*) was utilized to estimate the trend in the pneumonia death by age.

Univariate logistic regression was performed first for all variables, and the results are presented as frequencies and percentages. The relationship between all adjusted confounders and mortality in different age groups was initially evaluated based on *P* values, odds ratios (ORs), and 95% confidence intervals (95% CIs). *P* < 0.05 was considered significant in all statistical tests. Variables with a *P* value <0.1 in the results of univariate logistic regression analysis were included in a multivariate logistic regression model to identify independent risk factors for death in pediatric patients with severe CAP by multiple stepwise regression. Variables with *P* < 0.1 were entered into the multivariate model, and those with a resulting *P* > 0.05 were then removed. The strength of association was estimated using adjusted ORs and 95% CIs. All statistical analyses were performed using SPSS version 23.0.

## Results

### Participant characteristics

From 2012 to 2019, there were a total of 21,220 children under 5 years of age admitted to the PICU, of whom 1961 (9.24%) were admitted with a diagnosis of CAP; 958 children with severe CAP were ultimately enrolled in our study as judged by screening, of whom 12 were not eligible due to an antimicrobial therapy duration <48 h and 1 was excluded because of imperfect data records. Ultimately, 945 children were recruited for analysis, including 341 young children aged 2–59 months, 47 infants aged 29 days to 2 months, and 557 neonates aged less than 28 days. Eighty-eight deaths occurred during the investigation, with an overall all-cause mortality rate of 9.3%. The Cochran-Armitage trend test showed that there was no significant difference between age and mortality (*Z* = 0.089, *P* = 0.929). The baseline features of the participants were shown in Table 1. In the group of neonates aged less than 28 days, 63.4% were male. Almost half of the neonates had low birth weight (42.0%), and most of those who died were underweight (75.0%) or had very severe pneumonia (75.0%). One hundred neonates (18.0%) received mechanical ventilation during hospitalization, including 21 nonsurvivors (21.0%). In the group of infants aged 29 days to 2 months, 55.3% were male. Most infants had normal birth weight (70.2%) and normal weight (66.0%), and more than half had very severe pneumonia (57.4%). In the group of children aged 2–59 months, 53.7% were male, and 62.5% had very severe pneumonia, resulting in more deaths. Young children with underweight accounted for 66.7% of the fatalities, and only 11 children (3.2%) received mechanical ventilation. The majority of severe CAP patients aged 0–59 months were hospitalized for 7–14 days.

**Table 1:** Characteristics of 945 infants and young children with severe CAP in PICUs.

Characteristics	0–28 days (*n* = 557)			29 days–2 months (*n* = 47)			2–59 months (*n* = 341)		
	Survivor (%)	Dead (%)	*P* value	Survivor (%)	Dead (%)	*P* value	Survivor (%)	Dead (%)	*P* value
	(*n* = 513)	(*n* = 44)		(*n* = 42)	(*n* = 5)		(*n* = 302)	(*n* = 39)	
Female gender	184 (35.9)	20 (45.5)	0.208	18 (42.9)	3 (60.0)	0.472	139 (46.0)	19 (48.7)	0.751
**Weight for age category**
Normal	306 (59.6)	11 (25.0)	Ref	29 (69.0)	2 (40.0)	Ref	204 (67.5)	13 (33.3)	Ref
Moderate underweight	77 (15.0)	9 (20.5)	0.272	1 (2.4)	1 (20.0)	0.404	49 (16.2)	9 (23.1)	0.610
Severe underweight	130 (25.3)	24 (54.6)	0.000	12 (28.6)	2 (40.0)	0.265	49 (16.2)	17 (43.6)	0.001
**Birth weight (kg)**
2.5–4	311 (60.6)	12 (27.3)	Ref	30 (71.5)	3 (60.0)	Ref	NA	NA	–
1.8–2.5	122 (23.8)	10 (22.7)	0.088	6 (14.3)	2 (40.0)	0.999	NA	NA	–
<1.8	80 (15.6)	22 (50.0)	0.000	6 (14.3)	0 (0.0)	0.236	NA	NA	–
Gestational age (GA) <33 (weeks)	79 (15.4)	18 (40.9)	0.000	5 (11.9)	0 (0.0)	0.999	NA	NA	–
Very severe pneumonia	147 (28.7)	33 (75.0)	0.000	23 (54.8)	4 (80.0)	0.303	183 (60.6)	30 (76.9)	0.052
Days with symptom ≥21^a^	NA	NA	–	NA	NA	–	30 (9.9)	8 (20.5)	0.054
Prior antibiotic treatment^b^	110 (21.4)	9 (20.5)	0.878	27 (64.3)	2 (40.0)	0.305	214 (70.9)	26 (66.7)	0.590
Febrile at PICU admission	7 (13.6)	1 (2.3)	0.631	2 (4.8)	0 (0.0)	0.999	37 (12.3)	11 (28.2)	0.009
Mechanical Ventilation	79 (15.4)	21 (47.7)	0.000	1 (2.4)	1 (20.0)	0.123	5 (1.7)	6 (15.4)	<0.001
White blood cell <5 or >15 (×10^9^/L)	226 (44.1)	25 (56.8)	0.105	8 (19.0)	1 (20.0)	0.959	66 (21.9)	15 (38.5)	0.024
**Length of hospitalization (days)**
0–6	48 (9.4)	27 (61.4)	Ref	11 (26.2)	4 (80.0)	Ref	72 (23.8)	17 (43.6)	Ref
7–14	333 (64.9)	12 (27.3)	0.000	27 (64.3)	1 (20.0)	0.999	211 (69.9)	17 (43.6)	0.003
>14	132(25.7)	5(11.4)	0.005	4(9.5)	0(0.0)	0.999	19(6.3)	5(12.8)	0.228

Note: Ref, reference; –, non estimable; NA, not available (the data for this program was missing a lot).

^a^ Prior to PICU admission.

^b^ During 24 h before PICU admission.

### Comorbidities

In the group of neonates aged less than 28 days, most of the patients had >3 diseases simultaneously (77.7%). The results revealed that a higher number of comorbidities significantly increased the risk of death (*P* = 0.01). CHD was the most common comorbidity, but no association was identified between CHD and severe CAP death (30.4% vs. 34.1%, *P* = 0.612). The secondary outcome was ARDS, which was statistically related to high mortality (26.5% vs. 59.1%, *P* = 0.00). In addition, death was more likely to occur in patients with combined anemia, pulmonary hypertension, encephalopathy, sepsis, neonatal asphyxia, hyperbilirubinemia, or gastrointestinal hemorrhage as shown in Table 2.

**Table 2:** Comorbidities and medications of 945 infants and young children with severe CAP in PICUs.

Characteristics	0–28 days (*n* = 557)			29 days–2 months (*n* = 47)			2–59 months (*n* = 341)		
	Survivor (%)	Dead (%)	*P* value	Survivor (%)	Dead (%)	*P* value	Survivor (%)	Dead (%)	*P* value
	(*n* = 513)	(*n* = 44)		(*n* = 42)	(*n* = 5)		(*n* = 302)	(*n* = 39)	
Number of comorbidities >3	391 (76.2)	42 (95.5)	0.010	17 (40.5)	2 (40.0)	0.984	151 (50.0)	37 (94.9)	<0.001
**Comorbidity**
Anemia	95 (18.5)	29 (65.9)	0.000	20 (47.6)	5 (100.0)	0.998	104 (34.4)	16 (41.0)	0.419
Congenital heart disease	156 (30.4)	15 (34.1)	0.612	21 (50.0)	5 (100.0)	0.998	178 (58.9)	27 (69.2)	0.22
Pulmonary arterial hypertension	13 (2.5)	8 (18.2)	0.000	6 (14.3)	2 (40.0)	0.171	84 (27.8)	17 (43.6)	0.045
Diarrhea	15 (2.9)	0 (0.0)	0.999	6 (14.3)	0 (0.0)	0.999	43 (14.2)	10 (25.6)	0.069
Encephalopathy	35 (6.8)	10 (22.7)	0.001	3 (7.1)	0 (0.0)	0.999	22 (7.3)	5 (12.8)	0.235
Sepsis	9 (1.8)	7 (15.9)	0.000	NA	NA	–	11 (3.6)	2 (5.1)	0.649
Acute respiratory distress syndrome	136 (26.5)	26 (59.1)	0.000	NA	NA	–	NA	NA	–
Neonatal asphyxia	20 (3.9)	7 (15.9)	0.001	NA	NA	–	NA	NA	–
Hyperbilirubinemia	88 (17.2)	13 (29.6)	0.044	1 (2.4)	0 (0.0)	1.000	NA	NA	–
Gastrointestinal hemorrhage	23 (4.5)	13 (29.6)	0.000	NA	NA	–	NA	NA	–
Number of initial antimicrobials ≥2	19 (3.7)	2 (4.5)	0.779	13 (31.0)	4 (80.0)	0.061	199 (65.9)	21 (53.8)	0.141
**Initial antimicrobial regimens**
Carbapenems	140 (27.3)	20 (45.5)	0.012	7 (16.7)	1 (20.0)	0.852	44 (14.6)	10 (25.6)	0.079
Carbapenems + antiviral drugs	1 (0.2)	0 (0.0)	1.00	2 (4.8)	0 (0.0)	0.999	49 (16.2)	5 (12.8)	0.585
Third-generation cephalosporins	55 (10.7)	2 (4.5)	0.21	15 (35.7)	0 (0.0)	0.998	54 (17.9)	7 (17.9)	0.992
Third-generation cephalosporins + antiviral drugs	5 (1.0)	0 (0.0)	0.999	9 (21.4)	4 (80.0)	0.023	136 (45.0)	14 (35.9)	0.282
Second-generation cephalosporins	281 (54.8)	20 (45.5)	0.236	6 (14.3)	0 (0.0)	0.000	3 (1.0)	0	0.991
Other β-lactams^a^	14 (2.7)	0 (0.0)	0.999	NA	NA	–	NA	NA	–
Others^b^	17 (3.3)	2 (4.5)	0.667	3 (7.1)	0 (0.0)	0.999	16 (5.3)	3 (7.7)	0.542
Grade higher than recommended by guidelines	163 (31.8)	22 (50.0)	0.069	20 (47.6)	5 (100.0)	0.046	245 (81.1)	32 (82.1)	0.567
**Concomitant medications**
Vasopressors	327 (63.7)	38 (86.4)	0.004	10 (23.8)	2 (40.0)	0.440	87 (28.8)	23 (60.0)	<0.001
Probiotics	402 (78.4)	18 (40.9)	0.000	18 (42.9)	0 (0.0)	0.998	101 (33.4)	15 (38.5)	0.534
Furosemide	212 (41.3)	26 (59.1)	0.025	27 (64.3)	5 (100.0)	0.998	200 (66.2)	38 (97.4)	0.004
Inhaled corticosteroids (ICS)	30 (5.8)	7 (15.9)	0.014	27 (64.3)	3 (60.0)	0.851	256 (84.8)	33 (84.6)	0.980
Corticosteroids	36 (7.0)	6 (13.6)	0.118	19 (45.2)	3 (60.0)	0.536	212 (70.2)	32 (82.1)	0.128
Bronchodilators	9 (1.8)	1 (2.3)	0.804	26 (61.9)	3 (60.0)	0.934	262 (86.8)	32 (82.1)	0.461
Ambroxol	175 (34.1)	25 (56.8)	0.003	17 (40.5)	2 (40.0)	0.984	189 (62.6)	19 (48.7)	0.098
Human immunoglobulin	44 (8.6)	12 (27.3)	0.000	19(45.2)	4(80.0)	0.174	171(56.6)	29(74.4)	0.038
Sedative-hypnotics	210(40.9)	25(56.8)	0.010	16(38.1)	4(80.0)	0.107	147(48.7)	27(69.2)	0.018

Note: Ref, reference; –, non estimable; NA, not available (the data for this program was missing a lot).

^a^ Piperacillin-tazobactam (*n* = 10), penicillin (*n* = 4).

^b^ 0–28 days: antifungal (*n* = 2), glycopeptides (*n* = 2), carbapenems + macrolides/macrolides + antiviral drugs (*n* = 5), third-generation cephalosporins + carbapenems/aminoglycosides (*n* = 6), second-generation cephalosporins + antiviral drugs + macrolides (*n* = 2), piperacillin-tazobactam + antiviral drugs + macrolides (*n* = 2); 29 days–2 months: macrolides (*n* = 1), second-generation cephalosporins + carbapenems/aminoglycosides (*n* = 2); 2–59 months: glycopeptides (*n* = 3), carbapenems + macrolides/glycopeptides (*n* = 7), third-generation cephalosporins + antiviral drug + macrolides/carbapenems (*n* = 8), antifungal + carbapenems (*n* = 1).

In the group of infants aged 29 days to 2 months, 40.4% of the patients had >3 diseases simultaneously. The most common comorbidities were anemia and CHD, but none of them were associated with mortality as shown in Table 2.

In the group of young children aged 2–59 months, mortality was also higher in patients with >3 combined diseases than in those with fewer comorbidities (*P* < 0.001). Similar to young infants, in young children, CHD was the most prevalent comorbidity among those with severe CAP. Compared with survivors, in non-survivors, a higher risk of death could result from severe CAP complicated with pulmonary hypertension as shown in Table 2.

### Antimicrobial treatment

The vast majority of initial antimicrobial treatments were empirical, owing to the difficulty in identifying pathogens; monotherapy with second-generation cephalosporin (54.0%), carbapenem (28.7%), third-generation cephalosporin (10.2%), and other β-lactams (2.5%) was the most frequent choice for neonates aged less than 28 days (95.5%), with a mortality rate of 7.8%. In the group of infants aged 29 days to 2 months, 30 patients were treated with monotherapy (63.8%), with third-generation cephalosporins being the most commonly used medications, and third-generation cephalosporins + antiviral drugs were most frequently used in combination therapy. However, over half of the children aged 2–59 months received ≥2 antimicrobials (64.5%), mainly antiviral drugs plus third-generation cephalosporin (68.2%) or carbapenem (24.5%). In the 121 patients who received single-drug therapy in this group, third-generation cephalosporin was the most universal choice (50.4%), followed by carbapenem (44.6%), and the mortality rate was as high as 14.9%, which was much higher than that of children who received ≥2 antimicrobials (9.5%). The multivariate analyses showed that there was no correlation between antimicrobial regimens and mortality (Table 2).

### Concomitant medications

As shown in Table 2, the concomitant medications used during hospitalization may also be associated with mortality. In the real world, other medications, including antiasthmatics, expectorants, immunotherapies, dietary supplements, diuretics, etc., were also widely employed in children with severe CAP in the PICU. Tabulated data indicated that children treated with sedative-hypnotics (40.9% vs. 56.8%, *P* = 0.01; 38.1% vs. 80.0%, *P* = 0.107; 48.7% vs. 69.2%, *P* = 0.018), furosemide (41.3% vs. 59.1%, *P* = 0.025; 64.3% vs. 100.0%, *P* = 0.998; 66.2% vs. 97.4%, *P* = 0.004) and vasopressors (63.7% vs. 86.4%, *P* = 0.004; 23.8% vs. 40.0%, *P* = 0.440; 28.8% vs. 60.0%, *P* < 0.001) had a higher mortality rate. Compared with the survivor group, a higher proportion of the non-survivor group received corticosteroids despite there being no significant correlation. In contrast, for neonates aged less than 28 days, there was a significantly lower mortality rate in those treated with probiotics (78.4% vs. 40.9%, *P* = 0.000). More survivors received ambroxol in the group aged 2–59 months (62.6% vs. 48.7%, *P* = 0.098).

### Microbiological findings

Of the 945 children, 41 (4.3%) had a positive blood culture and the great majority (73.2%) had only one pathogen detected. Gram-negative bacteria (29.3%) and fungi (29.3%) were the most prevalent pathogens in children with severe CAP in the PICU. Among them the majority was Enterobacteriaceae and Candida spp. Atypical bacteria ranked second (24.4%), followed by gram-positive bacteria (22.0%) and viruses (22.0%). Streptococcus pneumoniae and respiratory syncytial virus (RSV) frequently cause pneumonia in children and are rarely detected in severe CAP patients in the PICU. However, Fisher's exact tests revealed that none of these pathogens was significantly associated with mortality in the PICU. (Table 3).

**Table 3:** Microbiological findings in blood culture of children with severe CAP in PICUs (*n* = 41).

Pathogens	Survivor (%) (*n* = 33)	Dead (%) (*n* = 8)	*P* values
**Gram-negative bacteria**	9 (27.3)	3 (37.5)	0.672
Acinetobacter baumannii	1 (3.0)	1 (12.5)	0.356
Klebsiella pneumoniae	1 (3.0)	0 (0.0)	1.000
Pseudomonas aeruginosa	1 (3.0)	0 (0.0)	1.000
Stenotrophomnas maltophilia	1 (3.0)	0 (0.0)	1.000
Enterobacteriaceae	5 (15.2)	2 (25.0)	0.606
**Gram-positive bacteria**	7 (21.2)	2 (25.0)	0.816
Staphylococcus	6 (18.2)	1 (12.5)	0.702
Streptococcus pneumoniae	1 (3.0)	1 (12.5)	0.356
**Atypical pathogens**	7 (21.2)	3 (37.5)	0.112
Mycoplasma spp.	5 (15.2)	2 (25.0)	0.606
Chlamydia spp.	2 (6.1)	1 (12.5)	0.488
**Fungus**	11 (33.3)	1 (12.5)	0.398
Candida spp.	11 (33.3)	1 (12.5)	0.398
**Virus**	8 (24.2)	1 (12.5)	0.472
Adenovirus	3 (9.1)	0 (0.0)	1.000
EB virus	4 (12.1)	1 (12.5)	0.977
Respiratory syncytial virus	1 (3.0)	0 (0.0)	1.000
**Number of pathogens**
1	24 (72.7)	6 (75.0)	0.896
≥2	9(27.3)	2(25.0)	0.078

### Independent risk factors for severe CAP death

In neonates aged less than 28 days, very severe pneumonia (OR: 3.48; 95% CI: 1.36–8.93), a birth weight <1.8 kg (OR: 4.64; 95% CI: 1.78–12.20) and mechanical ventilation use (OR: 2.58; 95% CI: 1.00–6.62) were identified as independent risk factors for death through multivariable logistic regression analysis adjusted for covariates. In addition, when severe childhood CAP was accompanied by anemia (OR: 5.24; 95% CI: 2.33–11.76) or gastrointestinal hemorrhage (OR: 3.79; 95% CI: 1.36–10.53), the mortality rate increased. Moreover, sedative-hypnotics (OR: 2.60; 95% CI: 1.14–5.95) were independently associated with a higher risk of death and probiotics were associated with a lower risk of death (OR: 0.14; 95% CI: 0.06–0.33) (Table 4).

**Table 4:** Risk factors independently associated with death from severe CAP.

Age groups	Factors	Adjusted Odds Ratio (95% CI)	*P* value
0–28 days	Very severe pneumonia	3.48 (1.36–8.93)	0.009
	Birth weight <1.8 (kg)	4.64 (1.78–12.20)	0.002
	Mechanical Ventilation	2.58 (1.00–6.62)	0.049
	Anemia	5.24 (2.33–11.76)	0.000
	Gastrointestinal hemorrhage	3.79 (1.36–10.53)	0.011
	Probiotics	0.14 (0.06–0.33)	0.000
	Sedative-hypnotics	2.60 (1.14–5.95)	0.024
2–59 months	Severe underweight	6.06 (2.34–15.63)	0.000
	Mechanical ventilation	15.63 (3.25–76.92)	0.010
	Number of comorbidities >3	8.40 (1.89–37.04)	0.005

In infants aged 29 days to 2 months, there were no independent risk factors screened by multivariable logistic regression analysis (Table 4).

In young children aged 2–59 months, a higher risk of death was independently associated with the following risk factors: severe underweight (OR: 6.06; 95% CI: 2.34–15.63); mechanical ventilation use (OR: 15.63; 95% CI 3.25–76.92); and a higher number of comorbidities (OR: 8.40; 95% CI: 1.89–37.04) (Table 4).

## Discussion

In our study, the incidence of CAP in children under 5 years of age admitted to the PICU was approximately 10%, with an incidence of WHO-defined severe CAP of approximately 50%; these children had a mortality rate as high as 9.3%, practically in line with that reported in a previous study with a larger cohort of 15,709 patients (10).

For neonates aged less than 28 days, the mortality rate was slightly lower at 7.9%. Several independent risk factors identified in our multivariate analyses as being associated with death were also observed in previous studies. Very severe pneumonia and lower birth weight were eminent mortality predictors of severe CAP (2, 11, 28). Thus, we should precisely distinguish the severity of CAP and weight categories for neonates at admission. Comorbidities played an important role in severe CAP death, and severe childhood pneumonia combined with anemia and gastrointestinal hemorrhage had a higher mortality rate in our study, similar to the results of a prospective cohort study carried out by Penelope et al. (10). Anemia is a common disease in childhood that is widespread in children in low- and middle-income countries. It is also a common comorbidity of severe pneumonia in children. Studies have reported that 45% of preschool children worldwide suffer from anemia, which increases to 65% in preschool children in Africa and Southeast Asia (30). Moschovis PP et al. published a study on the correlation between clinical treatment outcomes and anemia in children with severe pneumonia in 2013, which showed that severe pneumonia was more difficult to treat in children with anemia and more likely to show clinical treatment failure because the cause of death in these children was usually one of the following: respiratory failure, severe hypoxemia at the terminal stage of severe pneumonia due to an abnormal ventilatory blood flow ratio, or inadequate oxygen being delivered to important organs of the body. Children with anemia have a decreased oxygen-carrying capacity of hemoglobin, aggravating the phenomenon of an abnormal ventilatory blood flow ratio, thereby increasing respiratory failure risk in those with severe pneumonia; therefore, anemia could significantly increase the treatment failure rate and mortality of children with severe pneumonia and should attract sufficient attention from doctors (4). Consequently, accurate diagnosis and active treatment are essential to reduce severe CAP mortality.

All forementioned correlations were as expected, and the extraordinary feature of this research was that detailed data on concomitant medications used during hospitalization in the real world were collected and analyzed. In the analysis for sedative-hypnotics, we excluded medications used for sedation in mechanically ventilated patients. Finally, there were only four sedative drugs, including phenobarbital, chloral hydrate, midazolam and diazepam, used in the research population. After adjusting for the severity of severe CAP and mechanical ventilation use, our multivariate results suggested that sedative-hypnotics were significantly related to increased mortality, which was consistent with earlier studies. In a systematic meta-analysis of 12 controlled trials including 982 infants, phenobarbital significantly increased the need for mechanical ventilation by inhibiting respiratory function, resulting in apnea and respiratory failure (22). In their retrospective analyses, Lützen et al. demonstrated that life-threatening respiratory depression occurred in patients with pneumonia who were treated with phenobarbital, despite the low probability of phenobarbital-induced respiratory insufficiency (31). Therefore, sedative-hypnotics are generally not recommended for severe childhood CAP. If necessary, arterial blood gas measurement should be performed regularly to monitor end-tidal CO_2_, and children are at risk of apnea when the end-tidal CO_2_ is <30 mmHg or >50 mmHg (32). Moreover, probiotics were independently associated with a reduced risk of death, which was similar to the result of a prospective multicenter RCT carried out in 9 NICUs in Colombia, in which the investigator observed a lower morbidity and mortality of nosocomial infection, including pneumonia, in the probiotic group (33). In other RCTs, Biswal et al. described that prophylactic probiotics could apparently decrease the incidence of ventilator-associated pneumonia in children in the PICU and NICU (34). This might be due to inhibiting the overgrowth of pathogens by rehabilitating nonpathogenic bacteria to compete with pathogens as well as optimizing local and systemic immunity. Moreover, colonization of probiotics in the gastrointestinal tract can reduce intestinal permeability and competitively restrain attachment of pathogens, thereby reducing the possibility of colonization and translocation. In conclusion, probiotics could decrease the incidence and all-cause mortality of nosocomial infections, including severe pneumonia (33, 35). On the other hand, diarrhea is very common in infants and young children, accounting for almost 10% of deaths, as shown in our results. A recent study showed that probiotics played a protective role in preventing diarrhea-related fatalities (36). Critically ill children could possibly benefit from probiotics. Therefore, the use of probiotics should be of particular concern in children with severe CAP in the ICU.

In the neonates aged less than 28 days in our study, most patients received second-generation cephalosporins or third-generation cephalosporins, which met guideline recommendations. There were also some neonates who received initial empirical regimens of carbapenems, and surprisingly, although not statistically significant, the mortality rate of patients who received higher levels of antibiotics was not reduced; that is, in the clinical treatment of severe CAP in neonates, the efficacy of antibacterial drugs with initial empirical selection grades higher than those recommended by guidelines was not better than that of antibacterial drugs with grades recommended by guidelines, and the former also increased the risk of pathogen resistance, which may lead to higher mortality (14). The same results were observed in infants aged 29 days to 2 months.

In terms of young children aged 2–59 months, comorbidities were also a risk factor that was closely associated with mortality, and death was more likely to occur in patients with a higher number of comorbidities. In our study, the mortality rate in children with severe CAP combined with **≥**4 comorbidities was 8 times higher than that in those with ≤3 comorbidities, which was consistent with previous reports (37, 38). Severe underweight was also strongly predictive of mortality. It is well known that the weight class and nutritional status of children are closely related, and severe underweight tends to predict poor nutritional status. Malnutrition seriously affects the cellular and humoral immune processes in children, resulting in decreased systemic immune function. In addition, malnutrition leads to gastrointestinal dysfunction and intestinal bacterial translocation, increasing the incidence of nosocomial infection and thereby increasing the all-cause mortality rate of children with severe pneumonia (39).

In our study, mechanical ventilation use was a stronger independent predictor for mortality both in neonates aged less than 28 days and in young children aged 2–59 months. However, we believed that children who received mechanical ventilation had higher mortality, which reflected acute conditions and the severity of severe pneumonia rather than the influence of mechanical ventilation on mortality. Length of hospitalization mostly were 7–14 days in severe CAP children aged 0–59 months, but most of the death cases had a shorter hospitalization time, occurring within 6 days of admission, with a mortality rate of 36% in neonates aged less than 28 days, 27% in infants aged 29days–2 months and 19% in young children aged 2–59 months, which was consistent with the results of a prospective cohort study enrolling 100 children with CAP conducted in Tanzania by Serena et al. showing that deaths usually occurred within 8 days of admission (40). Children with severe pneumonia who stayed within 6 days had a significantly higher mortality rate than those who stayed ≥7 days, as with mechanical ventilation, which did not mean that shorter length of hospital stay was a risk factor for death from severe pneumonia in children, and that death within 6 days of admission only reflected the more serious condition of the patient.

A wide range of extended spectrum antimicrobials, including carbapenems, carbapenems + antiviral drugs and third-generation cephalosporins + antiviral drugs, was widely selected as initial empirical antimicrobial regimens in critically ill children aged 2–59 months with severe CAP, which revealed low adherence to guidelines; in the multivariate model, no significant difference in efficacy was observed between the guideline-recommended antimicrobials and others. Therefore, initial antimicrobial regimens for severe childhood CAP should be chosen based on the WHO classification of severity and guideline-recommended treatment consistent with the severity, which may promote rational antimicrobial use for severe childhood CAP and consequently prevent antimicrobial resistance.

Despite adopting detailed information from the real world and rigorous criteria, our research has several limitations. First, our retrospective study might have bias to some extent. Variables that were not documented in the medical records but are potentially associated with death could not be analyzed, including vaccination status, breastfeeding duration, adverse drug reactions, oxygen saturation and some inflammation marker levels. Second, a few comorbidities, such as sepsis or anemia, diagnosed by physicians lacked laboratory evidence, which significantly affected our results. Therefore, it could not be decided whether these conditions were misdiagnosed. The last limitations are the small sample size, especially in the group of infants aged 29 days–2 months, restricting the capacity to adjust for confounders, and that no statistically significant independent risk factors were obtained. We will continue to supplement and improve the data in the future.

## Conclusions

In conclusion, 9.3% of the children with severe CAP in the PICU died, and those with very severe pneumonia, severe underweight or lower birth weight were at a higher risk of death. Gram-negative bacteria are prevalent in severe childhood CAP. In addition, there is a significant influence of concomitant medications (e.g., sedative-hypnotics and probiotics) and comorbidities on clinical outcomes. Severe CAP is a primary cause of death in children, and particular attention should be given to identifying mortality predictors and establishing prophylactic measures to reduce mortality.

## Data Availability

The raw data supporting the conclusions of this article will be made available by the authors, without undue reservation.
